# A TOX-ic axis of epigenetic stem cell maintenance and chemoresistance in colon cancer

**DOI:** 10.1371/journal.pbio.3002295

**Published:** 2023-09-15

**Authors:** Christopher G. Hubert, Shaun R. Stauffer, Justin D. Lathia

**Affiliations:** 1 Department of Biochemistry, Case Western Reserve University, Cleveland, Ohio, United States of America; 2 Case Comprehensive Cancer Center, Cleveland, Ohio, United States of America; 3 Lerner Research Institute, Cleveland Clinic, Cleveland, Ohio, United States of America; 4 Rose Ella Burkhardt Brain Tumor & Neuro-Oncology Center, Cleveland Clinic, Cleveland, Ohio, United States of America

## Abstract

Cancer stem cells drive tumor growth and survival via self-renewal and therapeutic resistance, but the upstream mechanisms are unclear. This Primer explores a PLOS Biology study in colon cancer that reveals a new signaling network linking epigenetic regulation to these phenotypes.

## Teaser

Cancer stem cells drive tumor growth and survival via self-renewal and therapeutic resistance, but the upstream mechanisms are not well defined. In this issue of *PLOS Biology*, a study in colon cancer reveals a new signalling network that links epigenetic regulation to these phenotypes.

**Fig 1:**
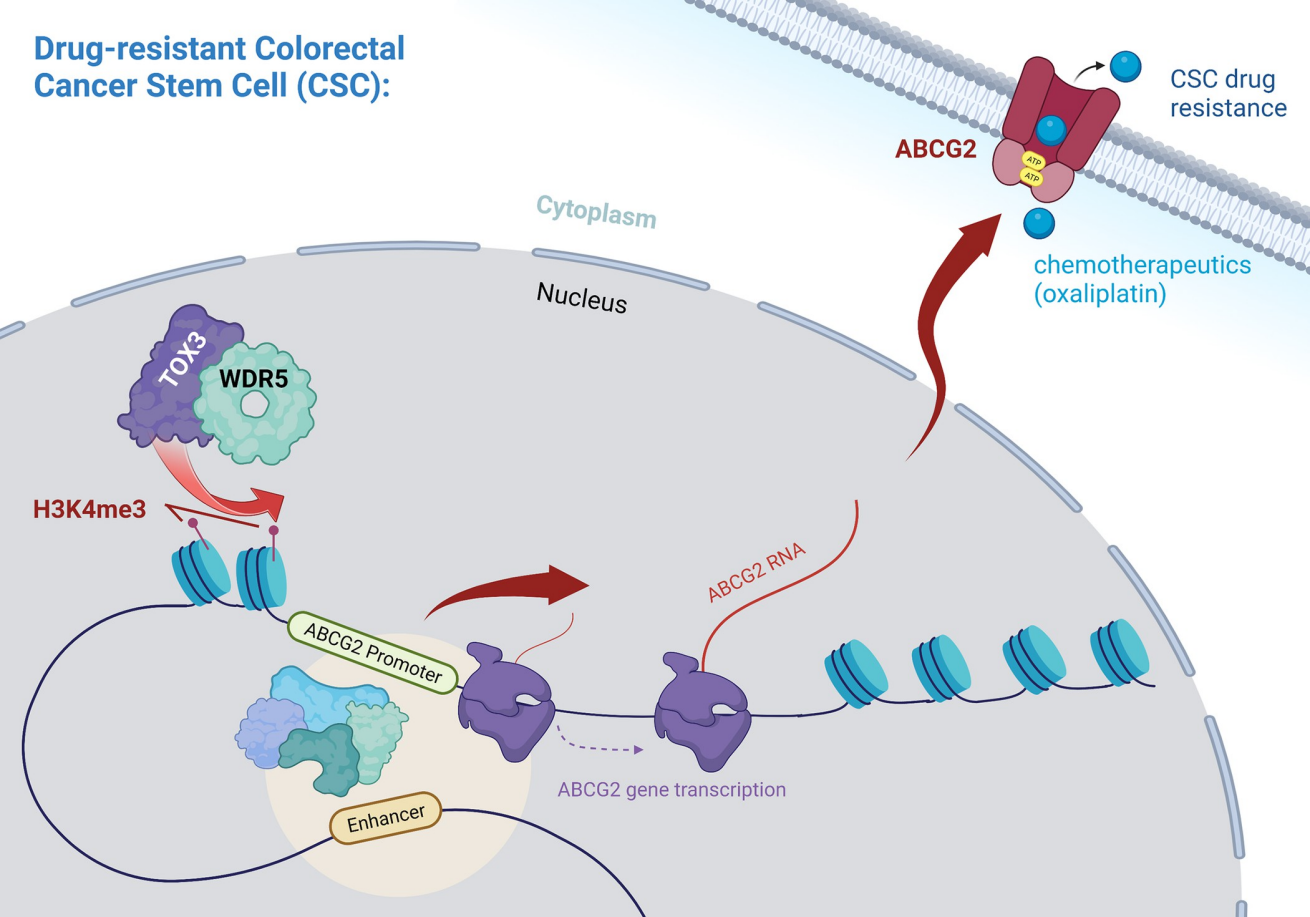
Proposed mechanism for functional drug resistance in colorectal cancer stem cells due to TOX3-WDR5 driven transcription of ABCG2. Interaction of TOX3 and WDR5 promotes pro-transcriptional H3K4me3 methylation at the promoter of the multidrug transporter ABCG2 in CRC CSCs. The increased protein levels of ABCG2 result in increased chemotherapeutic drug resistance through active drug efflux, a hallmark of cancer stem cells in many tumor types. Created with BioRender.com.

Cancer stem cells (CSCs) have been identified in multiple tumors and sustain tumor growth through self-renewal programs [1]. In addition, CSCs are generally refractory to standard of care radiation and chemotherapies used for many cancers [2–4]. Given their importance in both tumor growth and therapeutic resistance, CSCs are an attractive therapeutic target for next-generation therapies [5]. To leverage this possibility, however, a detailed understanding of the molecular mechanisms driving CSC maintenance is required. While well-known cell-intrinsic programs drive self-renewal via pluripotency transcription factors, there are fewer mechanisms identified that underly therapeutic resistance and very few potentially ideal therapeutic targets that regulate both phenotypes at once. Adding to the complexity of CSC regulation is that many factors important for these phenotypes are also essential for normal somatic stem cell populations, limiting the development of targeted therapies. Recent work has identified an essential role for epigenetics in regulating CSC maintenance and the epigenetic state is different between CSCs and somatic stem cells, offering a putative target for therapeutic development [6]. The missing piece of knowledge is exactly how key CSC phenotypes are driven by the epigenetic state. In this issue of *PLoS Biology*, Hao and colleagues [7] revealed that the transcription factor TOX3 regulated ABCG2, an efflux transporter known to drive chemoresistance, in colon cancer. TOX3 recruited WD repeat containing protein 5 (WDR5), a key epigenetic CSC factor, to promote trimethylation on the ABCG2 promoter at histone 3 lysine 4 (H3K4), thus activating its transcription. This work demonstrates a new signalling axis linking the epigenetic state to self-renewal and chemoresistance (Fig 1).

The Guo lab identified this mechanism through an integrated experimental approach that used human CSC models, gain/loss of function and rescue studies, and validation in a colitis-based tumor model and human datasets [7]. This manuscript provides a key link between self-renewal and therapeutic resistance programs, from a key cell surface drug efflux pump to a transcription factor and epigenetic state. There are limited examples of this type of detailed signalling network in CSCs, including a report on CD55 in gynecological malignancies [8], and such a detailed signalling axis provides many access points for targeting as the authors have shown with genetic perturbations of TOX3, ABCG2, WDR5, and the use of a small molecule WDR5 inhibitor. While these findings provide much needed insight into the complex interactions between the epigenetic state and CSC phenotypes, this study raises several questions.

For their assessments, Hao and colleagues used a system in which colon cancer cells were grown in CSC conditions and further enriched for chemotherapy-resistant CSC via addition of oxaliplatin [7]. This platform has known limitations in that the starting population was a high-passage cell line that is less representative of the CSC state and the cellular heterogeneity present in low-passage patient-derived models. The authors demonstrated functional validation for the enrichment of CSCs; however, confirmation of the molecular mechanisms identified here in low-passage patient-derived models with a direct comparison between CSCs and their non-CSC progeny represents a future priority. The researchers validated their findings in human datasets, where there was an association with poor prognosis, suggesting increased patient relevance despite the inherent drawbacks of the particular culture model system. In the future, it will be useful to assess this new signalling network in the context of normal stem cells from the colon to assess how specific TOX3, ABCG2, and WDR5 are to CSCs, which would help inform future therapeutic development efforts. Our scientific understanding of CSCs has been evolving and it is now appreciated that there can be many populations of CSCs with distinct properties, including different proliferative, metabolic, and developmental profiles. It would be interesting as assess how this newly identified signalling network operates in the context of CSC heterogeneity, as the epigenetic state is likely to reflect and potentially even drive such heterogeneity in cancers.

The connection between chemoresistance and the epigenetic state revealed a role for WDR5, which controls H3K4 trimethylation and generally functions as an activating mark [7]. WDR5 is a member of the WRAD complex, which contains other proteins including retinoblastoma binding protein 5 (RbBP5), absent-small-homeotic-2-like protein (ASH2L), and Dumpy-30 (Dpy30), and interacts with mixed-lineage leukemia 1 (MLL1) to control the epigenetic state [9]. Interestingly, many individual components of the WRAD complex have been shown to be important in glioblastoma CSCs, namely WDR5 [10], RBBP5 [11], and Dpy30 [12]. Given this, it would be interesting to see if any of these other WRAD complex components are important in this newly defined signalling axis. Of specific interest could be if any other components are recruited by TOX3 versus if this interaction was specific just to TOX3 and WDR5. Insight into the precise molecular interactions is likely to inform future drug development efforts.

To complement their genetic perturbations, the Guo lab leveraged a tool compound that inhibits WDR5, OICR-9429 [7]. OICR-9429 was first disclosed from the Structural Genomics Consortium (SGC) and the Drug Discovery Program at the Ontario Institute for Cancer Research (OICR) in 2015 [13]. The molecule is a first generation non-peptidic WDR5 WBM-site small molecule probe that binds within the central arginine cavity of WDR5 that recognizes the ARA motif found in MLL and other WBM site-binding partners. OICR-9429 is a selective probe binding to WDR5 with a K_d_ of 93 nM using SPR and a demonstrated displacement of MLL peptide with similar potency (K_disp_ = 65 nM). By current standards, this is a weak WDR5 WBM binder and thus newer molecules with >100× improvements in affinity are now reported and could be employed to further interrogate the TOX3-WDR5 interaction and signalling axis. OICR-9429’s weaker potency creates the need to use relatively high concentrations of inhibitor in cell-based studies reported by the authors. In contrast, the dose used for in vivo studies here is comparatively low. Overall, newer, more potent, and selective tool compounds will be valuable for future studies. Importantly, these newer potent chemotypes have demonstrated target engagement in cells and have matched negative control compounds that are pharmacologically inactive at WDR5.

As with many good scientific studies, this work serves as a starting point to initiate many follow up studies. While colon cancer was used for these assessments, there are many other tumors that are driven by CSCs and it would be useful to assess how these other tumors may leverage this new signalling network to drive self-renewal and chemoresistance, especially since many members of the WRAD complex are known to be essential in GBM CSCs as well. Another major question that these studies raise is the extent to other epigenetic marks are impacted by ABCG2-TOX3-WDR5, including other methylation marks and possibly other epigenetic modifications (acetylation, etc.). Finally, as CSCs represent attractive next-generation targets, the extent to which ABCG2-TOX3-WDR5 can be leveraged for therapeutic development should be an immediate priority. This includes the assessment of ABCG2-TOX3-WDR5 in other cancers, as mentioned above, as well as in tissue resident somatic stem cells, and leveraging ongoing efforts on the development of WDR5 inhibitors. Taken together, Hao and colleagues [7] provide evidence that self-renewal and chemoresistance, hallmarks of the CSC state involve complex molecular networks and identify a new series of putative anticancer targets ripe for exploitation in the future.
